# Physician reported outcomes of hip arthroscopy without a perineal post: an international survey

**DOI:** 10.1093/jhps/hnac038

**Published:** 2022-07-26

**Authors:** Alexander Volpi, Kristin Twomey Hopkins, Malachy McHugh, Gregory Galano

**Affiliations:** Lenox Hill Hospital, Nicholas Institute of Sports Medicine and Athletic Trauma, 210 E 64th St 5th floor, New York, NY 10065, USA; Lenox Hill Hospital, Nicholas Institute of Sports Medicine and Athletic Trauma, 210 E 64th St 5th floor, New York, NY 10065, USA; Lenox Hill Hospital, Nicholas Institute of Sports Medicine and Athletic Trauma, 210 E 64th St 5th floor, New York, NY 10065, USA; Lenox Hill Hospital, Nicholas Institute of Sports Medicine and Athletic Trauma, 210 E 64th St 5th floor, New York, NY 10065, USA

## Abstract

Although the current literature reports an acceptable rate of complications with the use of a perineal post in hip arthroscopy, they are still possible and preventable. The purpose of this study was to survey International Society for Hip Arthroscopy (ISHA) members on their use of postless distraction in hip arthroscopy. A 19-question survey was emailed to hip preservation surgeons that are members of ISHA. The questions examined surgeons’ location, experience, utilization of a perineal post or postless distraction and any complications they may have encountered. In all, 145 respondents completed the survey. Regarding complications encountered when using a perineal post, the most frequent responses were temporary nerve damage (115, 80.6%), temporary genitourinary complications (39, 27%), temporary genital skin injury (35, 24%) and permanent nerve injury (12, 8%). Regarding the postless technique, of the 60 respondents who noted they have utilized postless distraction, 9 (15%) reported complications, with 7 (12%) reporting temporary nerve damage being the most common and 0 reporting cases of permanent nerve injury. These were statistically significantly less than those reported with a perineal post. Ninety-seven percent reported that after utilizing postless distraction, their patients were recovering better than or the same as when using a perineal post. This survey had excellent international participation by experienced hip arthroscopists. There were a statistically significantly decreased number of complications reported by the surgeons utilizing postless distraction. This survey highlights that postless distraction is being done successfully with lower reported complications and excellent patient recovery.

## INTRODUCTION

Hip arthroscopy is a rapidly developing and growing procedure in orthopedics, as the understanding of hip pathology in the young athletic population evolves. In the literature, an overall acceptable complication rate of approximately 1.5% has been reported; however, the rates can vary [1–3]. The most commonly reported complications include those related to traction, fluid management, bony complications and surgical instruments [1, 3]. In order to access the central compartment of the hip, traction must be applied to the operative leg in order to pass instruments and perform intra-articular work. Traditionally, hip arthroscopy is done in the supine or lateral position, and a padded perineal post has been utilized to provide countertraction [4]. Complications related to traction and use of a perineal post are relatively common and are a result of compression of the perineum. In 1992, Glick first reported 4 transient neurapraxias of the pudendal nerve out of 60 cases [2]. Several years later, Funke and Munzinger reported pudendal nerve injury but also a hematoma of the labia majora, which was attributed to poor padding of the perineal post [5]. Additionally, Griffin and Villar reported a small vaginal tear that healed uneventfully [6]. Sampson reported 20 neurapraxias in a series of 1000 patients; 14 of the transient neuropraxias involved either the perineal or pudendal nerves [7]. Byrd reported a complication rate of 1.34% after roughly 1500 hip arthroscopies, including 6 pudendal neuropraxias and 1 case of scrotal necrosis [1]. Although the literature demonstrates that complications in hip arthroscopy are rare and have been decreasing over time, when they occur, it is commonly due to the traction post pressure in the perineum [3].

The senior author of this study has had recent success without the use of a perineal post when performing hip arthroscopy. Instead, a ‘Pink Pad’, commonly used in bariatric surgery, along with several degrees of Trendelenburg position, has been able to provide adequate countertraction without the use of a perineal post. In the cases where a perineal post has not been used, adequate traction has been achieved, patients report no groin pain or numbness and patients have overall felt better in the early postoperative period. The purpose of this study was to survey members of the Hip Preservation Society [International Society for Hip Arthroscopy (ISHA)] on their experience of hip arthroscopy with and without a perineal post and complications that they have encountered.

## METHODS

A 19-question, Institutional Review Board (IRB)-exempt, Health Insurance Portability and Accountability Act (HIPAA)-compliant survey was conducted using Research Electronic Data Capture (see Supplementary Data). The survey was sent to the ISHA administration, which was then distributed to their membership via email after being approved by their research committee. A total of two follow-up emails were sent over several weeks. The survey was not endorsed by any regional, national or international medical, surgical or hip preservation society.

Five questions were asked about surgeons’ training, location and experience. The remaining 14 questions were focused on complications and the use of postless distraction when performing hip arthroscopy.

Statistical analysis was done using chi-square analysis for linear association. Comparisons were made between surgeon experience and case volume, as well as surgeon experience and utilization of postless distraction. Reported complications were then compared between use of a perineal post and postless arthroscopy. Statistical significance was reported as a *P* value less than 0.05.

## RESULTS

A total of 145 respondents of the 610 ISHA members answered the survey. Surgeons from 29 countries on 6 continents completed the survey, including the United States, Brazil, the United Kingdom, Japan, South Africa and Australia. Regarding demographic data, when asked about experience level, 77 respondents (53.1%) reported >10 years’ experience, 40 (27.6%) with 5–10 years, 20 (13.8%) with 2–5 years and 8 (5.5%) with <2 years’ experience. The level of training in orthopedic surgery was reported as 67 (46.2%) completing only residency, 83 (57.2%) sports medicine fellowship, 60 (41.4%) hip preservation fellowship, 39 (26.9%) arthroplasty fellowship, 11 (7.6%) pediatric orthopedics fellowship, 1 (0.7%) pediatric sports medicine fellowship and 12 (8.3%) completing another form of training not listed. When surveyed on the number of hip arthroscopies performed in their career, 83 (57.6%) reported >500 cases, 12 (8.3%) reported 401–500 cases, 34 (23.6%) reported 100–400 and 15 (10.4%) reported <100 cases. As a follow-up, the number of hip arthroscopies performed in the last 12 months were 10 (6.9%) >350 cases, 37 (25.7%) 151–350 cases, 43 (29.9%) 100–150 cases and 54 (37.5%) <100 cases.

Regarding complications encountered, respondents were asked to select complications they have encountered when using a perineal post for distraction during hip arthroscopy. Responses included 115 (80.6%) temporary nerve damage, 12 (8.3%) permanent nerve damage, 39 (27.1%) temporary genitourinary complications, 0 permanent genitourinary complications, 35 (24.3%) temporary genital skin injury, 2 (1.4%) permanent genital skin injury and 19 (13.2%) selected other. As a follow-up question, the rate at which these reported complications had occurred was asked. Seventy-four (51.4%) reported Rare (<1% of the time), 42 (29.4%) selected Uncommon (1–10%), 12 (8.4%) chose Common (>10% of the time) and 15 (10.5%) chose Other.

Regarding postless distraction, 60 (41.7%) respondents stated that they have used postless distraction, with 38 (28.6%) reporting that they currently use postless distraction. Those respondents reported performing an average of 156 ± 301 (SD, range 0–1500) postless arthroscopies. Regarding which postless table device was used by surgeons, 37 (27.6%) reported Stryker Guardian table, 25 (18.7%) selected Pink Pad with Smith and Nephew Table, 6 (4.5%) chose Pink Pad with Hana Table, 11 (8.2%) chose other, and 70 (52.2%) selected N/A (not applicable). Fifty-three (38.7%) reported that they used Trendelenburg positioning during postless, with a reported average of 14° (SD ±5, range 7.5–30).

In addressing the learning curve of postless distraction, 30 (50%) reported <5 cases needed to be comfortable with postless distraction, 14 (23%) needing 5–10 cases, 5 (8%) needing 11–20 cases and 0 requiring >20 cases. Eighty-five (63%) reported that 0% of their hip arthroscopy cases were performed with postless distraction, 13 (9.6%) selected 1–32% of cases, 1 (0.7%) chose 33–66%, 3 (2.2%) chose 67–99% of cases and 33 (24.4%) reported 100% of cases being done without a perineal post.

Regarding the complications reported with postless distraction, there were 7 (12%) reported cases of temporary nerve damage, 0 of permanent nerve damage, 1 (2%) of temporary genitourinary complications, 0 of permanent genitourinary complications, 1 (2%) of temporary genital skin injury and 0 of permanent genital skin injury. Seventeen (12.4%) respondents noted that they have had to abandon postless distraction and use a perineal post. The most frequent response to this question is inadequate distraction. Finally, in being polled as to how patients having undergone postless distraction recover compared to those after using a perineal post, 47 (57.3%) replied the Same, 33, (40.2%) selected Better and 2 (2.4%) Worse.

On chi-square analysis, investigating surgeon experience and case volume, more experienced surgeons reported doing a higher volume of cases annually and in their careers total (*P* < 0.0001). When looking at the effect of surgeon experience and if they have ever used postless distraction, it was noted that prior use of postless distraction was more prevalent in the less experienced surgeons (*P* < 0.0001); only 26% of surgeons with more than 10 years experience reported having used postless distraction versus 74% of the surgeons with 10 or fewer years experience. However, the number of lifetime cases did not appear to influence prior use of postless distraction (*P* = 0.827); 39% of surgeons who had performed more than 500 hip arthroscopies reported having used postless distraction versus 44% for surgeons who had performed fewer cases. Moreover, prior use of postless arthroscopy was more prevalent in surgeons with a higher current annual case volume (*P* = 0.047); 52% of surgeons who performed more than 150 hip arthroscopies in the previous year reported using postless distraction versus 36% of surgeons with fewer cases in the previous year.

When examining the current use of postless distraction, only 12% of surgeons with more than 10 years’ experience were using postless distraction compared with 43% for less experienced surgeons (*P* < 0.0001). The number of lifetime cases (*P* = 0.246) and the number of cases in the previous year (*P* = 0.492) were unrelated to current use of postless distraction. Regarding whether respondents have had to abandon postless distraction (and convert to a perineal post), surgeon experience, number of lifetime cases and number of annual cases were unrelated to abandonment of the postless distraction technique. Of the surgeons who reported having previously used postless distraction, 40% reported having to abandon postless traction setup and switch to a perineal post.

**Fig. 1.**
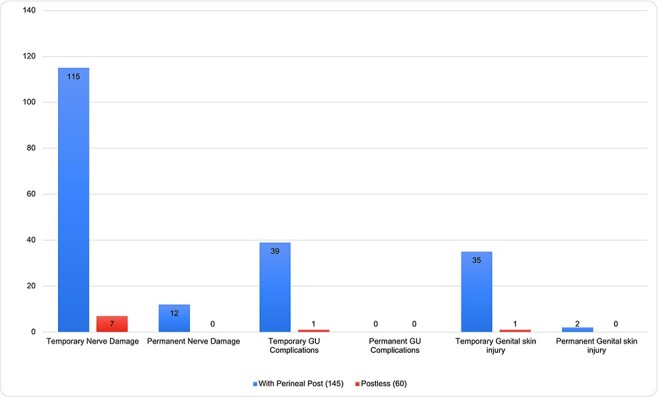
Comparison of reported postoperative complications when using a perineal post versus postless technique.

For the analysis of reported complications, comparing postless to post distraction, the number of respondents reporting cases of temporary nerve damage was significantly lower with postless technique: 7 of 60 (12%) versus 115 of 145 (80%) using a perineal post (*P* = 0.0001). No respondents reported any cases of permanent nerve damage with the postless technique (0 of 60, 0%) versus 12 of 145 (8%) respondents reported cases of permanent damage with the perineal post (*P* = 0.020). For temporary genitourinary complications, there were 1 of 60 (2%) postless versus 39 of 145 (27%) when using a post (*P* < 0.0001). No respondents reported permanent genitourinary complications with either technique. For temporary genital skin injury, 1 in 60 (2%) were reported using postless versus 35 of 145 (24%) reported using a post (*P* < 0.0001). For permanent genital skin injury, there were 0 of 60 (0%) reported using postless versus 2 of 145 (1%) using the perineal post (*P* = 0.999). The summary of comparisons of reported complications is found in Fig. 1.

**Fig. 2.**
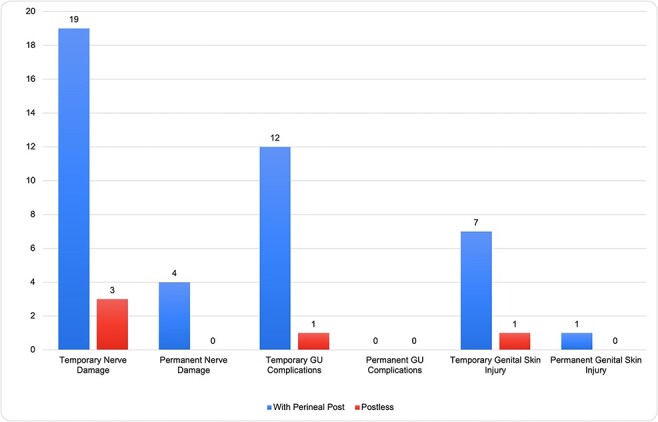
Subgroup analysis of high-volume, experienced surgeon’s reported postoperative complications when using a perineal post versus postless technique.

A subgroup analysis was performed by examining surgeons who had greater than 5 years of experience and performed more than 150 hip arthroscopies per year. There were 43 surgeon respondents of this experience and volume level, of which 20 reported using postless distraction. In all, 19 of 20 (95%) of these high-volume, experienced surgeons reported temporary nerve damage when using a post compared to only 3 of 20 (15%) when using the postless technique (*P* < 0.001). Regarding permanent nerve damage, 4 of 20 (20%) surgeons reported this complication using the post, while 0 reported this complication with the postless technique (*P* < 0.011). Twelve of 20 (60%) surgeons reported temporary genitourinary complications with the perineal post compared to 1 of 20 (5%) with the postless technique (*P* = 0.004). No surgeons reported permanent genitourinary complications with either technique. Regarding temporary genital skin injury, 7 of 20 (35%) surgeons reported this complication while using a post compared to 1 of 20 (5%) reported with the postless technique (*P* = 0.0436). Finally, only 1 surgeon reported permanent genital skin injury while using a perineal post. A summary of these results is found in Fig. 2.

## DISCUSSION

Overall, there was an excellent response to this international survey with a total of 145 physicians participating from around the world. This survey drew several main subjective conclusions from the respondents. The survey responses showed that less experienced surgeons and those with a higher annual case volume were utilizing postless arthroscopy. Less than half of the survey respondents noted ever using postless distraction and less than one-quarter currently uses postless distraction. Secondly, on the analysis of reported complications, comparing postless to post distraction, the number of respondents reporting cases of temporary nerve damage, temporary genitourinary complications, permanent nerve damage and temporary genital skin injury was statistically significantly lower with postless technique. These trends were also seen on our further subgroup analysis of experienced, high-volume surgeons. Thirdly, when polled as to how patients having undergone postless distraction are recovering compared to those after using a perineal post, roughly 97% replied the ‘Same’ or ‘Better.’

This study highlights the complications that can be encountered when using a perineal post for distraction, and the possible decrease in these complications if postless distraction is utilized. In a series of nearly 1500 patients, of the 20 complications (1.34%) Byrd reported, 7 were related to traction against the perineal post, including 6 cases of temporary pudendal nerve palsy [1]. Several other studies with more than 1000 patients also described roughly 1.5–2% of complications, noting that most of these complications were temporary neuropraxias involving various nerves from femoral, sciatic to perineal and pudendal [4, 8]. When studying the outcomes of postless hip arthroscopy, Mei-Dan reported that one of the co-authors at their institution performed 2000 hip arthroscopies without a perineal post and observed no groin or perineal complications [3]. A low rate of temporary nerve injury is reported in the literature, and the current survey shows that surgeons who utilize a perineal post for distraction encounter complications infrequently, with more than 50% of respondents reporting complications occurring in <1% of cases. However, what is a more striking takeaway of this survey is that 80% of responders noted encountering temporary nerve injuries with the use of a perineal post for distraction at some point in their practice, compared to a statistically significant difference of 12% reported with the postless technique. Although the frequency of reported complications parallels the current literature, these survey responses show that these types of complications may be encountered when using a perineal post. Of note, the rates of reported overall neurapraxia and nerve injury have decreased over recent years in the literature.

Soft tissue injury and genitourinary injury are also possible and preventable complications when using a perineal post. Of the previously mentioned studies and reported complications associated with a perineal post, each paper cites several groin and perineal complications including scrotal necrosis, vaginal tears, labia hematomas and soft tissue pressure necrosis to the perineum [1, 5, 8–10, 12]. As expected, when removing the perineal post, there is a lower risk for direct pressure injuries to the perineum, and this is reported in our current survey. Temporary genitourinary complications were reported at 27% in those that used a post compared to a statistically significantly lower 2% in the postless group. Temporary genital skin injury was reported similarly, with 24% in the post group and 2% in the postless group, also statistically significant. No respondents reported permanent genitourinary complications with either technique, and only one respondent in the post group reported a permanent genital skin injury.

This survey also highlighted some other correlations between surgeon experience and use of the postless distraction. Surgeon experience and the learning curve in hip arthroscopy have been studied in the literature. Go et al. published a systematic review looking at surgeon experience and complication rate [11]. They found a broad learning curve reported, ranging from 20 to 500 cases, and that factors such as operative time, complication rate and revision rate all decreased with surgeon experience. As expected, more experienced surgeons were performing more hip arthroscopies annually; however, less experienced surgeons made up the most responses utilizing postless distraction. Regarding the learning curve of postless distraction, half of the responders (30 of 60) noted that they were comfortable with the postless technique after <5 cases. Additionally, surgeon experience or case volume was unrelated to whether or not surgeons had to abandon the postless technique if they had tried it.

There were several limitations to this study. Firstly, this is purely a survey for hip arthroscopists, asking them to report on their own experiences. Answers and responses are based on surgeon memory, may be subject to recall bias or may be a rough estimation of their actual experiences and outcomes of hip arthroscopy. Secondly, although every respondent received the full survey and had the option to answer every question, not every respondent answered all questions, since some questions may not have pertained to each survey respondent. Although this changed the raw numbers for several questions, we did our best to analyze the data and drew pertinent conclusions from the responses given for each question. The number of respondents for each question is listed in the Supplemental Data. Finally, because this is a survey, there is no reporting of actual techniques or clinical outcomes, again with responses only based on anecdotal information.

Since this is the lowest level of evidence research, the results should be interpreted with caution as all data were reported on a subjective basis. Further directions of study with randomized controlled trials comparing distraction techniques with reported clinical outcomes would aid in demonstrating the growing use of postless distraction as well as its benefits regarding possibly decreasing postoperative complications. Additional postoperative complications such as cartilage injury and clinical outcome scores would also be beneficial to address in future studies. It should also be noted that postless distraction may not be suited for every patient and surgeon, as 40% of our respondents reported having to abandon postless distraction. Evaluation of patient and pathology type, surgeon skill and available equipment should be exercised prior to utilizing postless distraction.

In summary, this international survey yielded an excellent overall participation and highlighted subjectively that postless distraction in hip arthroscopy is increasing in use. When comparing the complications reported, there was a statistically significantly decreased number of complications reported by the surgeons utilizing postless distraction overall than those when using a perineal post. Although not a widely used technique, this survey highlights that postless distraction in hip arthroscopy is being done successfully with lower reported complications and excellent reported patient recovery. The surgeons who participated in the survey and reported their use of postless distraction showed that hip arthroscopy may be successfully performed without a perineal post, does not require a steep learning curve and can potentially lead to a decrease in complications and improve overall outcomes.

## Supplementary Material

hnac038_Supp
