# Supplementary material for: Physician reported outcomes of hip arthroscopy without a perineal post: an international survey
Source: J Hip Preserv Surg. 2022 Jul 26;11(1):3–7. doi: 10.1093/jhps/hnac038 (PMC11005764; doi:10.1093/jhps/hnac038)
Supplement: hnac038_Supp [file hnac038_supp.zip › Postless Supplemental Data Updated.docx]

**Supplementary Data**

**Postless Hip Arthroscopy Survey**

1. How many years have you been performing arthroscopic hip preservation surgery?
   1. <2yrs
   2. 2-5 yrs
   3. 5-10yrs
   4. >10yrs

Respondents n=145

1. What level of training in orthopaedic surgery have you completed? (Select all that apply)
   1. Residency
   2. Sports medicine fellowship
   3. Hip preservation fellowship
   4. Arthroplasty fellowship
   5. Pediatric Orthopedics fellowship
   6. Pediatric sports medicine fellowship
   7. Other

Respondents n=145

1. In what country do you currently practice? (SurveyMonkey/REDCAP to populate choices)

Respondents n=145

1. Approximately how many arthroscopic hip preservation surgeries have you done in your career?
   1. 0-100
   2. 100-400
   3. 400 – 500
   4. >500

Respondents n=145

1. Approximately how many arthroscopic hip preservation surgeries did you perform for FAI and/or labral tears over the last 12 months?
   1. <100
   2. 100 – 150
   3. 150 – 300
   4. >300

Respondents n=145

1. What complications have you encountered in hip arthroscopy when using a perineal post? (SELECT ALL THAT APPLY)
   1. Temporary nerve damage
   2. Permanent nerve damage
   3. Temporary genitourinary complications
   4. Permanent genitourinary complications
   5. Temporary genital skin injury
   6. Permanent genital skin injury
   7. n/a

Respondents n=145

7. If you selected one or more complications in the previous question, how often are these complications occurring?

1. Rare (<1%)
2. Uncommon (1-10%)
3. Common (>10%)

Respondents n=143

8. Have you ever used postless hip distraction?

a.Yes

b. No

Respondents n=145

9. Do you currently use postless distraction?

1. Yes
2. No

Respondents n=145

10. What have you utilized for postless distraction? Select all that apply

1. Stryker Guardian
2. Pink Pad with Hana table
3. Pink Pad with Smith and Nephew table
4. Other
5. N/a

Respondents n=134

11. Approximately how many hip arthroscopies have you performed using postless distraction?

Enter number

Respondents n=128

12. Do you use Trendelenburg position with your postless technique?

1. Yes
2. No

Respondents n=137

13. If you answered Yes to the previous question, how many degrees of Trendelenburg approximately do you use?

Enter Number

Respondents n=67

14. How long did it take you to become comfortable with postless distraction?

1. <5 cases
2. 5 – 10 cases
3. 10 – 20 cases
4. >20 cases
5. n/a

Respondents n=133

15. What percentage of your hip arthroscopy cases are being done with postless distraction?

1. 0%
2. 1 – 32%
3. 33 – 66%
4. 67 – 99%
5. 100%

Respondents n=135

16. What complications have you encountered using postless distraction for hip arthroscopy? SELECT ALL THAT APPLY

1. Temporary nerve damage
2. Permanent nerve damage
3. Temporary genitourinary complications
4. Permanent genitourinary complications
5. Temporary genital skin injury
6. Permanent genital skin injury
7. n/a

Respondents n=132

17. Have you had to abandon the postless technique and use a perineal post on any cases?

1. Yes
2. No
3. n/a

Respondents n=137

18. If you answered Yes to the previous question, why did you need to abandon the postless technique and use a perineal post on any cases?

Free text

Respondents n=24

19. In your opinion, are your patients who had postless distraction recovering (compared to traditional post distraction)

1. The same
2. Better
3. Worse

Respondents n=82
